# Supplementary material for: Selective catalytic two-step process for ethylene glycol from carbon monoxide
Source: Nat Commun. 2016 Jul 5;7:12075. doi: 10.1038/ncomms12075 (PMC4935967; doi:10.1038/ncomms12075)
Supplement: Supplementary Information — Supplementary Figure 1, Supplementary Tables 1-14 and Supplementary Methods [file ncomms12075-s1.pdf]

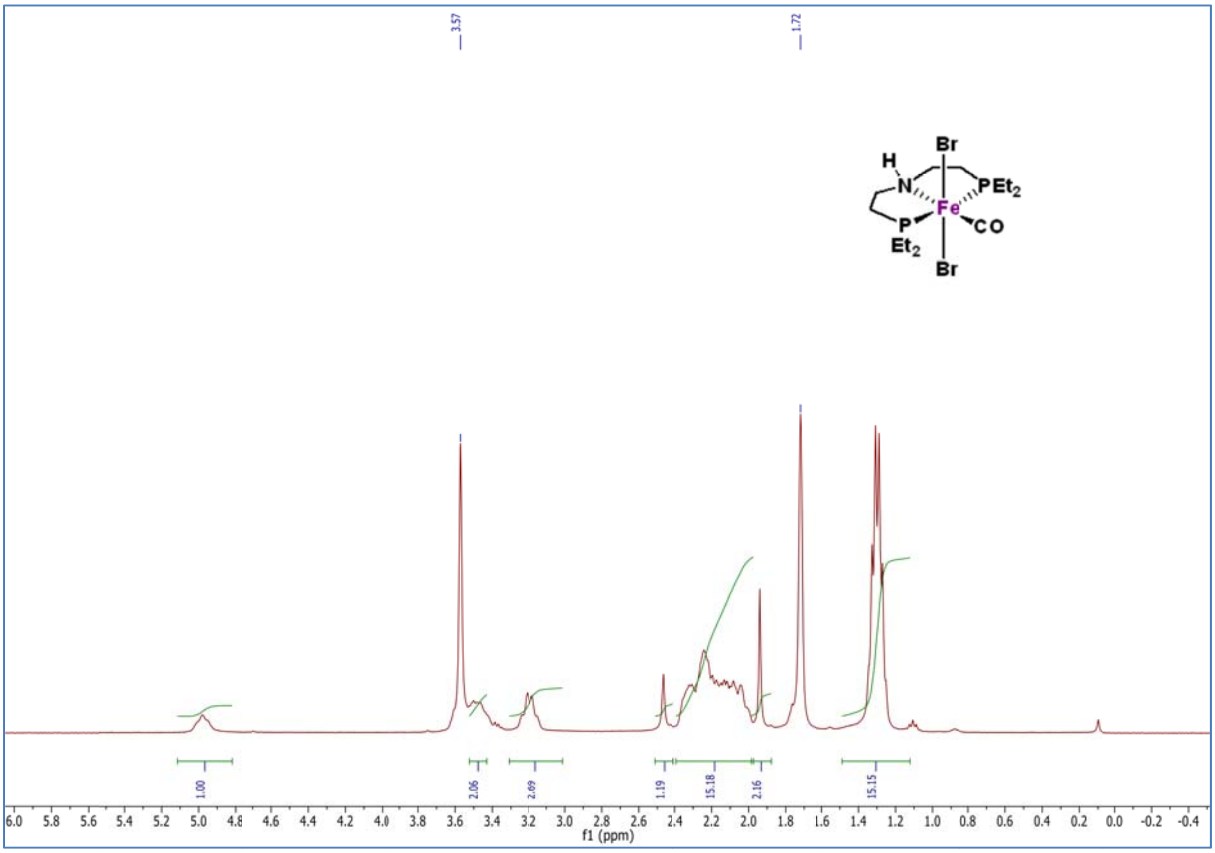
3.57
1.72
Br
H
N
PEt2
Fe
P
CO
Et2
Br
1.00
2.06
2.09
1.19
15.18
2.16
15.15
6.0 5.8 5.6 5.4 5.2 5.0 4.8 4.6 4.4 4.2 4.0 3.8 3.6 3.4 3.2 3.0 2.8 2.6 2.4 2.2 2.0 1.8 1.6 1.4 1.2 1.0 0.8 0.6 0.4 0.2 0.0 -0.2 -0.4
f1 (ppm)

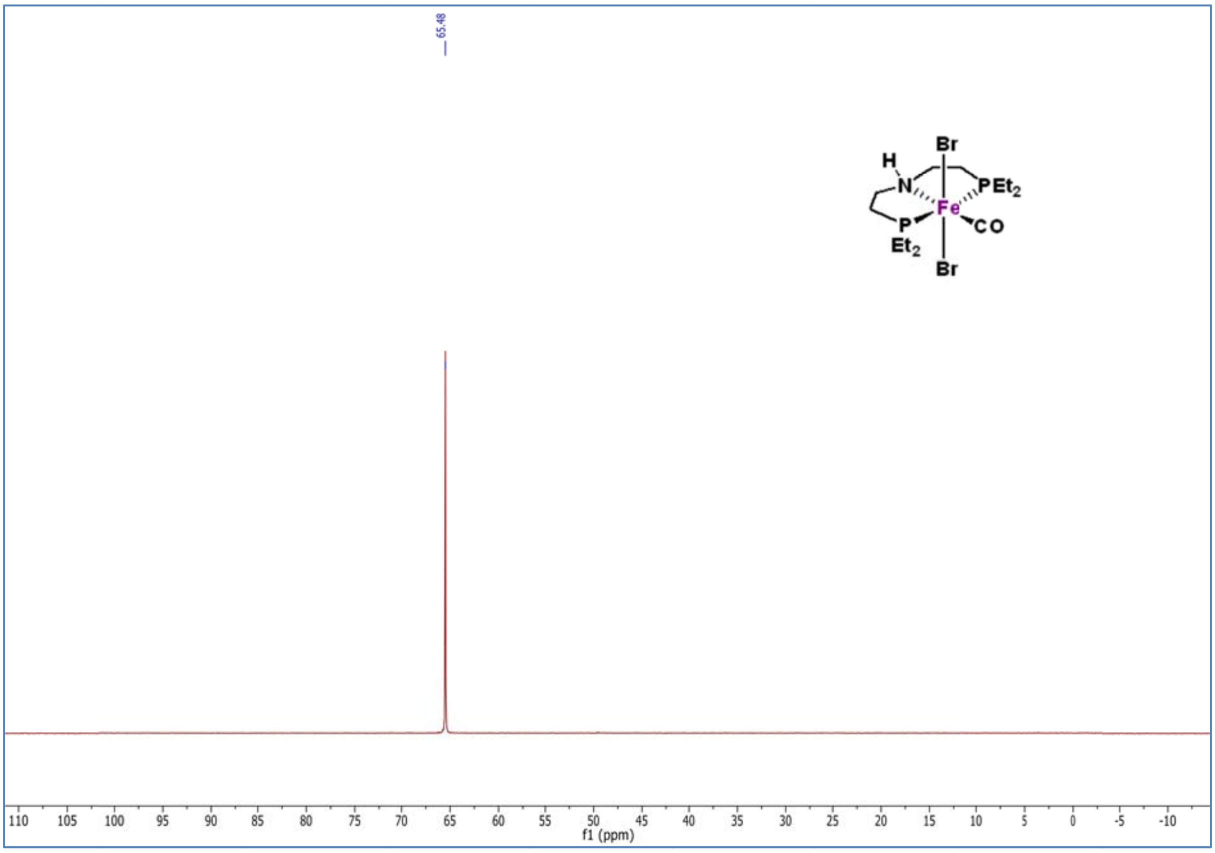
65.48
Br
H
N
PEt2
Fe
P
CO
Et2
Br
110 105 100 95 90 85 80 75 70 65 60 55 50 45 40 35 30 25 20 15 10 5 0 -5 -10
f1 (ppm)

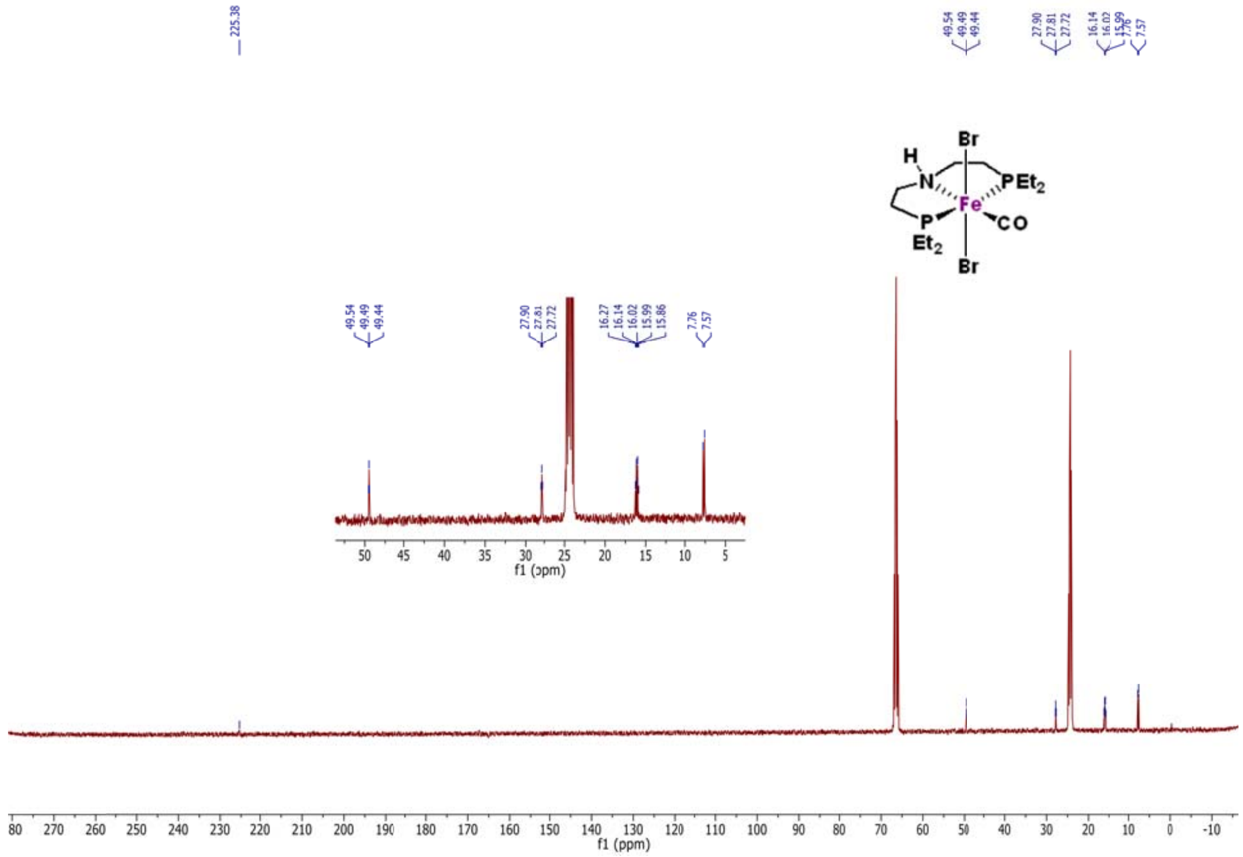

225.38
49.54
49.49
49.44
27.90
27.81
27.72
16.14
16.02
15.99
7.76
7.57
Br
H
N
PEt2
Fe
P
CO
Et2
Br
16.27
15.86
f1 (ppm)

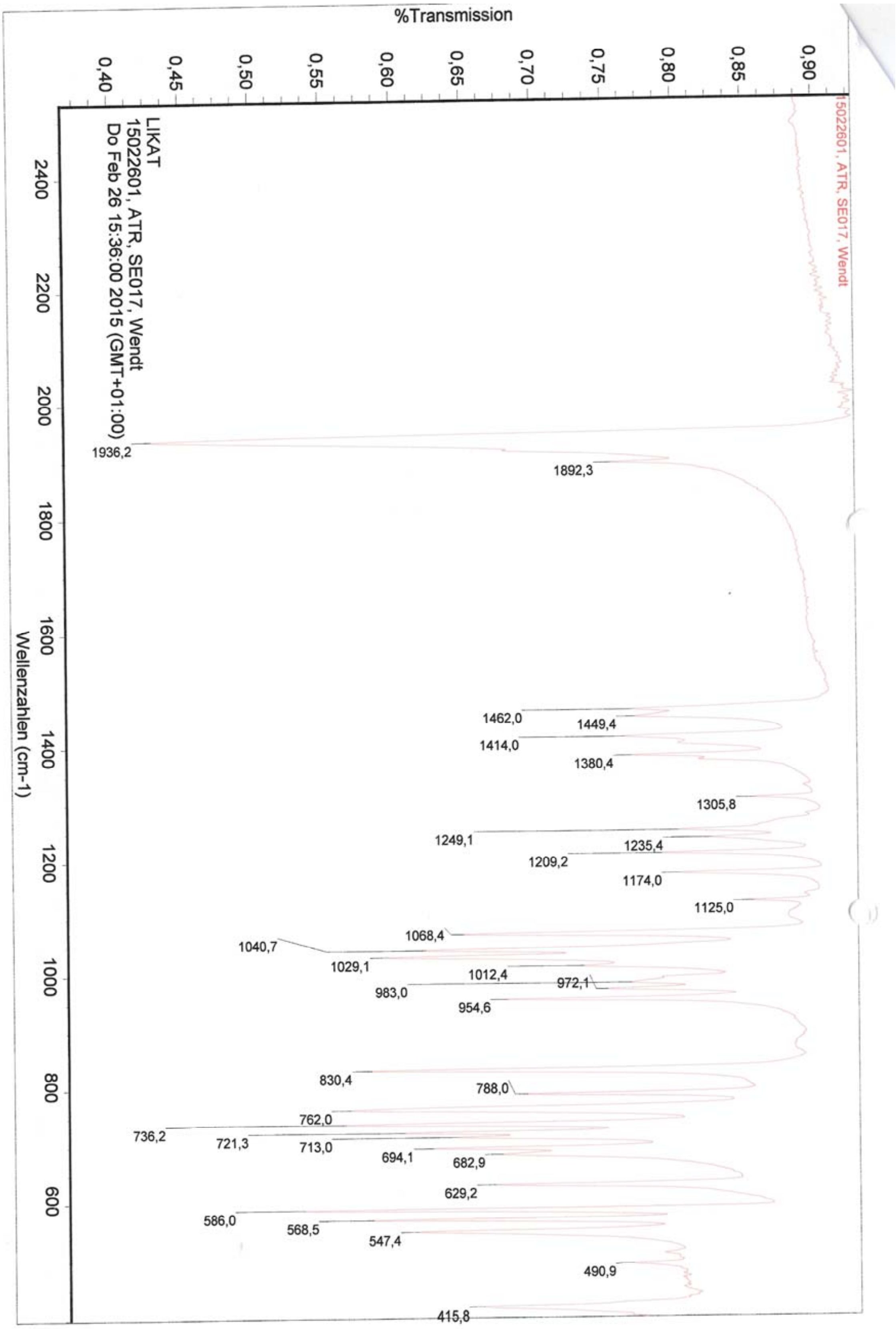

Supplementary Figure 1. ^{1}H, ^{31}P, ^{13}C NMR spectra, and ATR –IR spectrum of complex c-8

Supplementary Table 1. Effect of Pd sources on the oxidative carbonylation of piperidine.

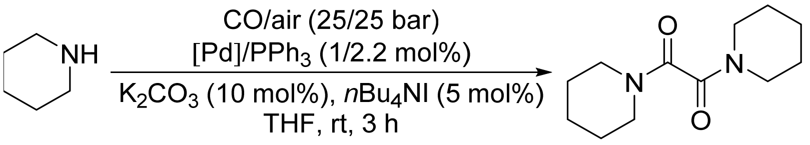

Entry	Pd source	Yield/%
1	$PdCl_2$	52
2	$Pd(OAc)_2$	53
3	$Pd(acac)_2$	54
4	$Pd(cod)Cl_2$	52

Reaction conditions: piperidine (100 uL, 1 mmol), CO/air (25 bar/25 bar), [Pd] (1 mol%), PPh_3 (5.8 mg, 2.2 mol%), K_2CO_3 (13.8 mg, 10 mol%), $n$$Bu_4NI$ (18.5 mg, 5 mol%), THF (2.0 mL), rt, 3h.

Supplementary Table 2. Effect of ligands on the Pd-catalyzed oxidative carbonylation of piperidine

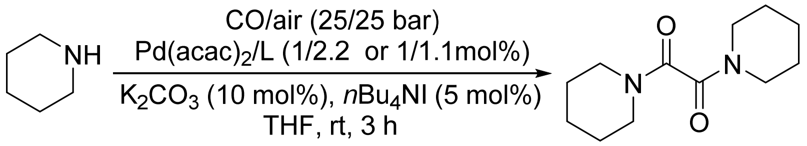

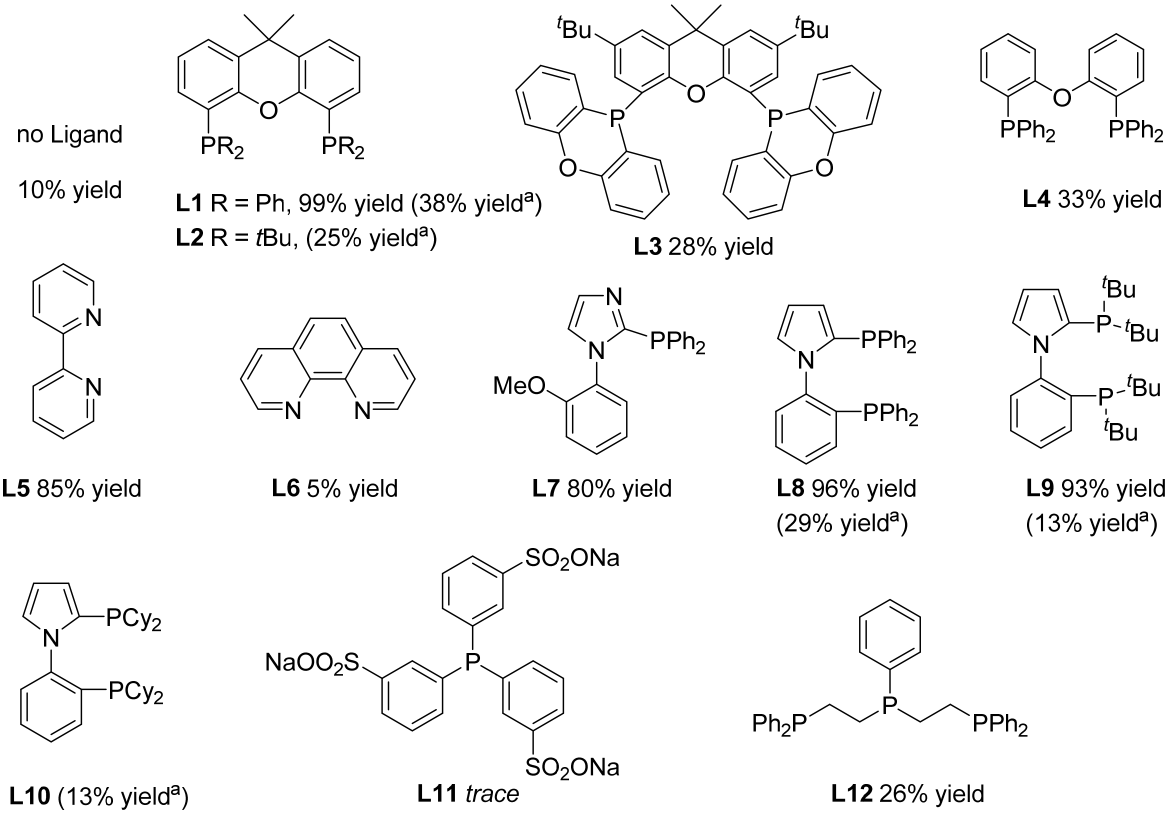

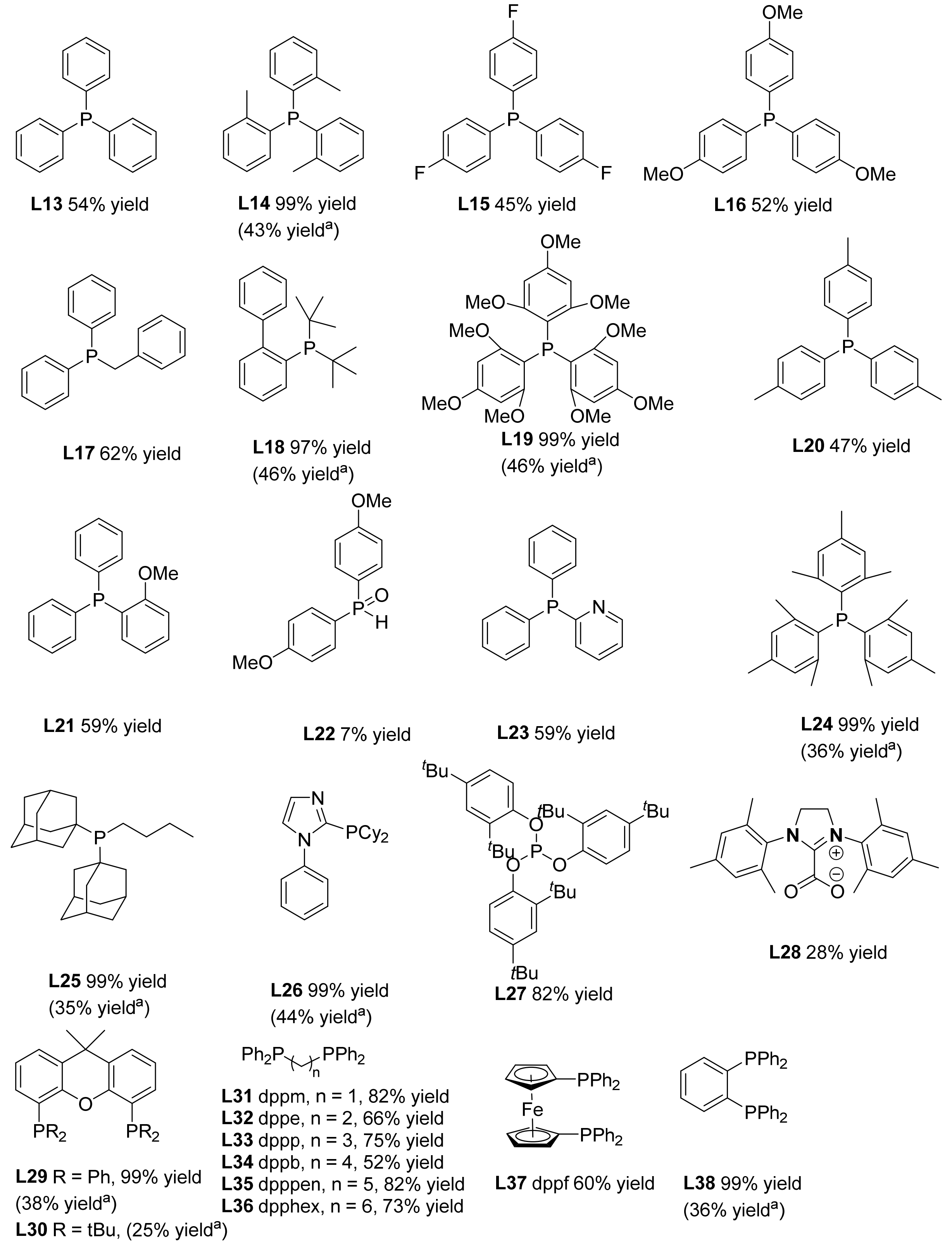

Reaction conditions: (1) 1 mol% Pd, piperidine (100 uL, 1 mmol), CO/air (25 bar/25 bar), $Pd(acac)_2$ (3.1 mg, 1 mol%), **L** (2.2 mol%), K_2CO_3 (13.8 mg, 10 mol%), *n*Bu_4NI (18.5 mg, 5 mol%), THF (2.0 mL), rt, 3h. (2) 0.2 mol% Pd, piperidine (250 uL, 2.5 mmol), CO/air (25 bar/25 bar), $Pd(acac)_2$ (1.5 mg, 0.2 mol%), monodentate ligand (0.44 mol%) or bidentate ligand (0.22 mol%), K_2CO_3 (69 mg, 10 mol%), *n*Bu_4NI (92 mg, 5 mol%), THF (5.0 mL), rt, 3h. [a] 0.2 mol% $Pd(acac)_2$.

Supplementary Table 3. Additive effect on the Pd-catalyzed oxidative carbonylation of piperidine

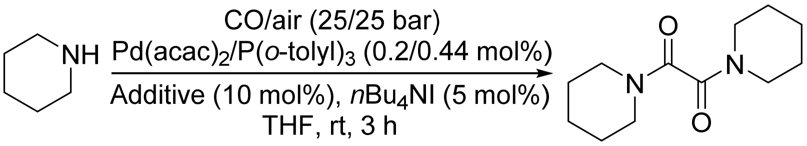

Entry	Additive (10 mol%)	Yield/%
1	^{t}BuOK	44
2	K_2CO_3 (10 mol%)	43
3	K_2CO_3 (5 mol%)	41
4	K_2CO_3 (20 mol%)	45
5	Cs_2CO_3 (10 mol%)	2
6	K_3PO_3 (10 mol%)	29
7	PTSA (10 mol%)	2
8	Al(OTf)$_3$ (10 mol%)	37

Reaction conditions: piperidine (250 uL, 2.5 mmol), CO/air (25 bar/25 bar), Pd(acac)$_2$ (1.5 mg, 0.2 mol%), P(*o*-tolyl)$_3$ (3.4 mg, 0.44 mol%), *n*Bu$_4$NI (92 mg, 5 mol%), THF (5.0 mL), rt, 3h.

Supplementary Table 4. Effect of iodine source on Pd-catalyzed oxidative carbonylation of piperidine.

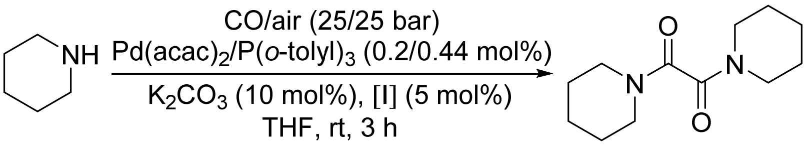

Entry	[I] (5 mol%)	Yield/%
1	*n*Bu$_4$NI (5 mol%)	43
2	*n*Bu$_4$NI (2 mol%)	39
3	*n*Bu$_4$NI (10 mol%)	46
4	NaI	*trace*
5	Et$_4$NI	5
6	CuI	7
7	I_2	*trace*

Reaction conditions: piperidine (250 uL, 2.5 mmol), CO/air (25 bar/25 bar), Pd(acac)$_2$ (1.5 mg, 0.2 mol%), P(*o*-tolyl)$_3$ (3.4 mg, 0.44 mol%), K_2CO_3 (69 mg, 10 mol%), THF (5.0 mL), rt, 3h.

Supplementary Table 5. Solvent effect on the Pd-catalyzed oxidative carbonylation of piperidine

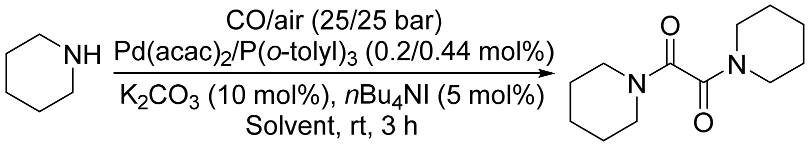

Entry	Solvent	Yield/%
1	THF	43
2	toluene	*trace*
3	MeOH	*trace*
4	CH_2Cl_2	*trace*
5	NMP	44
6	DMF	29
7	CH_3CN	3
8	dioxane	5

Reaction conditions: piperidine (250 uL, 2.5 mmol), CO/air (25 bar/25 bar), Pd(acac)$_2$ (1.5 mg, 0.2 mol%), P(*o*-tolyl)$_3$ (3.4 mg, 0.44 mol%), K_2CO_3 (69 mg, 10 mol%), *n*Bu$_4$NI (92 mg, 5 mol%), solvent (5.0 mL), rt, 3h.

Supplementary Table 6. Concentration effect on the Pd-catalyzed oxidative carbonylation of piperidine

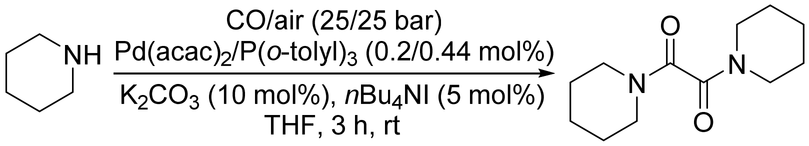

Entry	THF	Yield/%
1	2 mL	19
2	5 mL	43
3	10 mL	40

Reaction conditions: piperidine (250 uL, 2.5 mmol), CO/air (25 bar/25 bar), Pd(acac)$_2$ (1.5 mg, 0.2 mol%), P(*o*-tolyl)$_3$ (3.4 mg, 0.44 mol%), K_2CO_3 (69 mg, 10 mol%), *n*Bu$_4$NI (92 mg, 5 mol%), THF, rt, 3h.

Supplementary Table 7. Catalyst loading effect on the Pd-catalyzed oxidative carbonylation of piperidine

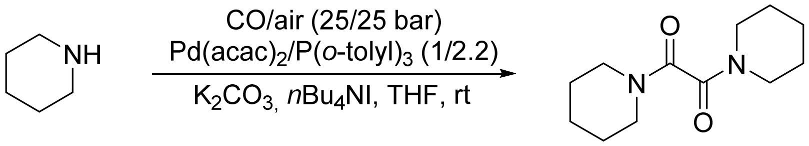

Entry	Catalyst loading	Time/h	Yield/%
1	1%	3	99
2	0.01%	50	92
3[a]	0.01%	4	60
4	0.001%	168	53

Reaction conditions: (1) 1 mol% Pd, piperidine (100 uL, 1 mmol), CO/air (25 bar/25 bar), $Pd(acac)_2$ (3.1 mg, 1 mol%), $P(o\text{-tolyl})_3$ (6.8 mg, 2.2 mol%), K_2CO_3 (13.8 mg, 10 mol%), $n\mathrm{Bu_4NI}$ (18.5 mg, 5 mol%), THF (2.0 mL), rt, 3h. (2) 0.01 mol% Pd: piperidine (5.0 mL, 50 mmol), CO/air (25 bar/25 bar), $Pd(acac)_2$ (1.52 mg, 0.01 mol%), $P(o\text{-tolyl})_3$ (61 mg, 0.4 mol%), K_2CO_3 (690 mg, 10 mol%), $n\mathrm{Bu_4NI}$ (925 mg, 5 mol%), THF (100 mL), rt, 50 h. (3) 0.001 mol% Pd: piperidine (5.0 mL, 50 mmol), CO/air (25 bar/25 bar), $Pd(acac)_2$ (0.152 mg, 0.001 mol%), $P(o\text{-tolyl})_3$ (183 mg, 1.2 mol%), K_2CO_3 (690 mg, 10 mol%), $n\mathrm{Bu_4NI}$ (925 mg, 5 mol%), THF (100 mL), rt, 168 h. [a] 120 ^{o}C, 4 h.

Supplementary Table 8. Ru-catalyzed hydrogenation of oxamide **2** to glycol

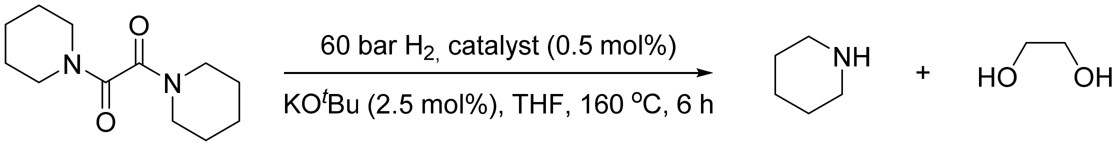

Entry	Catalyst	Conversion. (%)	piperidine (%)	Glycol (%)
1	Ru-MACHO-BH	20	10	5
2	Milstein catalyst	100	97	94
3[a]	Milstein catalyst	91	88	80
4[b]	Milstein catalyst	100	98	96
5	Noyori catalyst	25	10	*trace*
6	Saundan catalyst	64	35	8

Reaction conditions: substrate (224 mg, 1 mmol), Ru catalyst (0.5 mol%), $KO^t\mathrm{Bu}$ (2.8 mg, 2.5 mol%), H_2 (60 bar), THF (2 mL), 160 ^{o}C, 6 h. [a] Milstein catalyst (0.1 mol%), 12 h. [b] 130 ^{o}C.

Supplementary Table 9. Ru-catalyzed hydrogenation of oxamide **2** to glycol: Solvent and additive effects

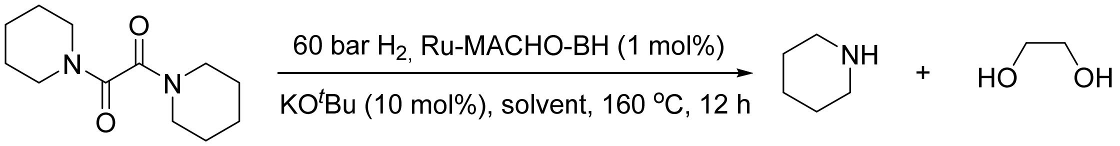

Entry	Solvent	Additive	Conversion. (%)	piperidine (%)	Glycol (%)
1	THF	--	60	50	40
2	THF	$PhNH_2$	94	92	88
3	THF	1*H*-imidazole	0	0	0
4	*i*PrOH	--	85	82	75
5	toluene	--	100	99	98
6	MeOH	--	25	22	19

Reaction conditions: substrate (224 mg, 1 mmol), Ru-MACHO-BH (1.0 mol%), KO^{t}Bu (11.2 mg, 10 mol%), H_2 (60 bar), solvent (2 mL), 160 °C, 12 h.

Supplementary Table 10. Ru-catalyzed hydrogenation of oxamide **2** to glycol: Base and solvent effects

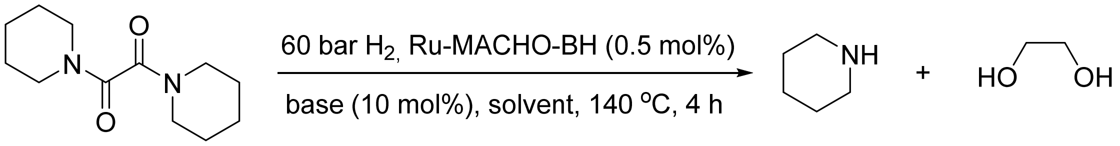

Entry	Solvent	Base	Conv./%	piperidine%	Glycol/%
1	Toluene	*t*BuOK (10 mol%)	60	55	53
2	Toluene	KOH (10 mol%)	83	78	75
3	Toluene	KOH (5 mol%)	80	70	65
4	Toluene	KOH (20 mol%)	84	78	75
5	Toluene	NaOH (10 mol%)	76	72	70
6	Toluene	Cs_2CO_3 (10 mol%)	80	72	68
7	Toluene	K_2CO_3 (10 mol%)	5	3	1
8	Toluene	$PhNH_2$ (1 eq)	0	0	0
9	Toluene	*t*BuOK (10 mol%) + $PhNH_2$ (1 eq)	55	45	40
10	Toluene	piperidine (1 eq)	0	0	0
11	*o*-xylene	KOH (10 mol%)	78	71	67
12	mesitylene	KOH (10 mol%)	88	81	78

Reaction conditions: substrate (224 mg, 1 mmol), Ru-MACHO-BH (1.0 mol%), base (10 mol%), H_2 (60 bar), solvent (2 mL), 140 °C, 4 h.

Supplementary Table 11. Ru-catalyzed hydrogenation of oxamide **2** to glycol: Base and additive effects

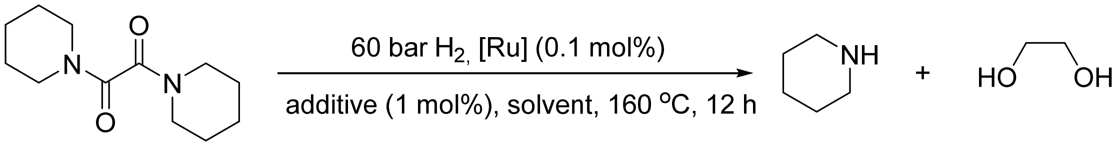

Entry	catalyst	additive	Conv./%	Yield (EG) /%
1	**c-11**	*t*BuOK (10 mol%)	67	63
2[b]	**c-11**	$Al(OTf)_3$ (2 mol%)	88	87
3	**c-11**	KOH (10 mol%)	88	78

Reaction conditions: substrate (224 mg, 1 mmol), Ru-MACHO-BH (1.0 mol%), base (10 mol%), H_2 (60 bar), solvent (2 mL), 140 °C, 4 h. b Mixture of toluene (1 mL) and water (1 mL) as solvent.

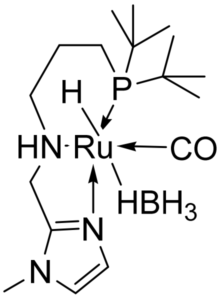

c-11

Supplementary Table 12. Ru-catalyzed hydrogenation of diphenyloxalamide to glycol with 1 mol% catalyst loading

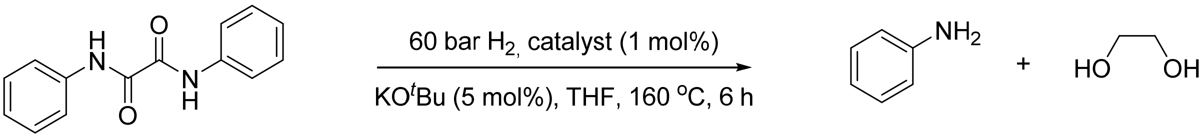

Entry	Catalyst	Conversion. (%)	Aniline (%)	Glycol (%)
1	Ru-MACHO-BH	100	99	99
2	Milstein catalyst	100	99	99
3[a]	$Ru(acac)_3$/triphos/$HNTf_2$	85	50	5
4[a]	$Ru(acac)_3$/triphos/$Al(OTf)_3$	96	52	5
5	Noyori catalyst	66	42	*trace*
6	Saudan catalyst	100	96	93
7	**c-3**	100	99	99

Reaction conditions: diphenyloxalamide (227 mg, 1 mmol), Ru catalyst (1 mol%), KO^{t}Bu (5.6 mg, 5 mol%), THF (2 mL), 160 °C, 6 h. [a] Ru/triphos/acid (1/1/5 mol%), no KO^{t}Bu.

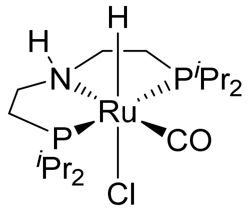

c-3

Supplementary Table 13. Ru-catalyzed hydrogenation of diphenyloxamide to glycol with 0.3-0.1 mol% catalyst loading

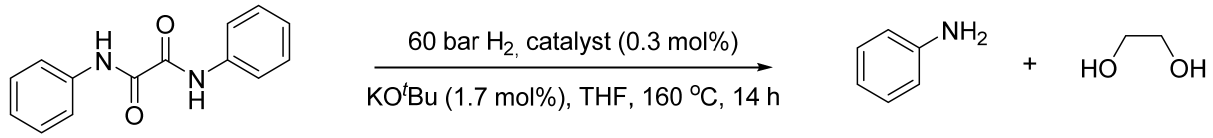

Entry	Catalyst	Conversion. (%)	Aniline (%)	Glycol (%)
1[a]	Ru-MACHO-BH	100	94	88
2	Ru-MACHO-BH	100	97	94
3	Milstein catalyst	100	96	92
4	Saudan catalyst	90	52	20
5[b]	Macho-Ru	90	55	10
6[b]	Milstein catalyst	81	51	10

Reaction conditions: diphenyloxalamide (227 mg, 1 mmol), Ru catalyst (0.3 mol%), KO^{t}Bu (5.6 mg, 5 mol%), H_2 (60 bar), THF (2 mL), 160 °C, 14 h. [a] Without KO^{t}Bu. [b] Ru catalyst (0.1 mol%), KO^{t}Bu (2.5 mol%).

Supplementary Table 14 Variation of reaction conditions in **step 2** using the reaction mixture of **step 1** as the starting material.

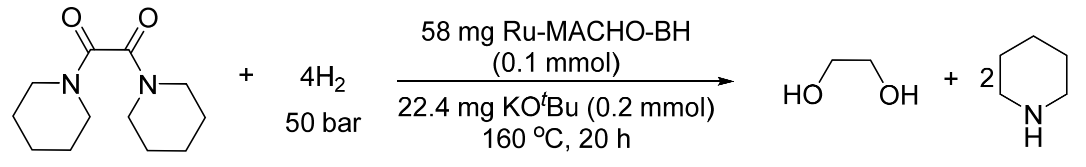

2
6 mL THF reaction mixture
from **step 1,** 5 mmol

3 **1**

Discussion: Notably, the hydrogenation of **2** in toluene is more efficient than in THF when using isolated **2** as the reactant. Therefore, we investigated the solvent effect with the reaction mixture of step 1 as the reactant (entry 1: THF, entry 2: THF/toluene, entry 3: toluene). Unfortunately, in all cases only low conversion of **2** was detected (5-28%). However, after short filtration (2-3 cm silica gel) to remove undissolved Pd particles EG was obtained in 98% yield as detected by ^{1}H NMR. When increasing the amount of the reaction mixture of step 1 from 6 mL to 20 mL, the reaction gave 92% yield of EG (entry 5).

Entry	Procedure for "one-pot" production of EG	Conv.(%)[a]	Yield/%[b]
1	After hydrogenation, 20 mL THF was added to 6 mL of the THF reaction mixture of step 1. Then, the newly resulting mixture was added to a 100 mL autoclave which was charged with 58 mg Ru-MACHO and 22.4 mg KO^tBu. After the autoclave was flushed with H_2 three times and pressurized with H_2 to 50 bar, the reaction was performed at 160 °C for 20 h.	26	--
2	After hydrogenation, 20 mL toluene was added to 6 mL of the THF reaction mixture of step 1. Then, the resulting mixture was added to a 100 mL autoclave which was charged with 58 mg Ru-MACHO and 22.4 mg KO^tBu. After the autoclave was flushed with H_2 three times and pressurized with H_2 to 50 bar, the reaction was performed at 160 °C for 20 h.	28	--
3	After hydrogenation, the solvent was removed in vacuo from 6 mL of reaction mixture of step 1 followed by addition of 20 mL toluene. The resulting mixture was stirred at RT for 20 min and then added to a 100 mL autoclave which was charged with 58 mg Ru-MACHO and 22.4 mg KO^tBu. After the autoclave was flushed with H_2 three times and pressurized with H_2 to 50 bar, the reaction was performed at 160 °C for 20 h.	5	--
4	After hydrogenation, THF was removed in vacuo from 6 mL of the reaction mixture of step 1 followed by addition of 20 mL toluene. The resulting mixture was stirred at RT for 20 min and then filtrated (silica gel, 2-3 cm). The filtrate was injected by syringe to a 100 mL autoclave which was charged with 58 mg Ru-MACHO and 22.4 mg KO^tBu. After the autoclave was flushed with H_2 three times and pressurized with H_2 to 50 bar, the reaction was performed at 160 °C for 20 h.	>99	98
5	After hydrogenation, THF was removed from 20 mL of the reaction mixture of step 1 followed by addition of 20 mL toluene. The resulting mixture was stirred at RT for 20 min and then filtrated through thin silica gel (2-3 cm). The filtrate was injected by syringe to a 100 mL autoclave which was charged with 58 mg Ru-MACHO and 22.4 mg KO^tBu. After the autoclave was flushed with H_2 three times and pressurized with H_2 to 50 bar, the reaction was performed at 160 °C for 20 h.	>99	92

a. Determined by GC using *iso*-octane as internal standard. *b.* ^1H NMR yield using *tert*-butanol as internal standard.

Supplementary Methods

General information

Air- and moisture-sensitive syntheses were performed under argon atmosphere in heating gun vacuum dried glassware. Chemicals were purchased from Aldrich, Alfa, Acros or Strem. Unless otherwise noted, all commercial reagents were used without further purification. ^{1}H and ^{13}C NMR spectra were recorded on Bruker Avance 300 (300 MHz) NMR spectrometers. Chemical shifts δ (ppm) are given relative to solvent: references for benzene-d_6 were 2.09 ppm (^{1}H-NMR) and 30.60 ppm (^{13}C-NMR). ^{13}C-NMR spectra were acquired on a broad band decoupled mode. GC analysis was performed on a Agilent 7890A chromatograph with a a 29 m HP5 column. The products were isolated from the reaction mixture by solvent evaporation and further purified by recrystallization in vacuum. Complexes c-1, c-2, c-3, c-4, c-5 (in situ) were purchased and used directly. Complexes **c-6**[28] and **c-10**[38] were prepared based literature methods.

General procedure for Pd-catalyzed oxidative carbonylation of piperidine

1) Under argon atmosphere, vial (4 mL) was charged with palladium precursor and ligand (monodentate ligand: Pd/**L** = 1/2.2, bidentate ligand: Pd/**L** = 1/1.1). The solvent was injected into the vial by the syringe and the solution was stirred for 10 min at room temperature. Then K_2CO_3, *n*Bu_4NI, and piperidine were added into the reaction solution. The vial was placed in an alloyed plate, which was then transferred into an autoclave (300 mL). At room temperature, the autoclave was pressurized with air (25 bar) and CO (25 bar). The reaction was performed at room temperature for desired time. After the reaction finished, the pressure was carefully released. The yield and selectivity were measured by GC analysis using isooctane as internal standard.

2) Consecutive experiments (4 runs): 8.5 g of amine **1** (0.1 mol) was used in 100 mL THF in the presence of 31 mg $Pd(acac)_2$ (0.1 mol%), 68 mg (*o*-tol)$_3$P (0.22 mol%), 0.9 g *n*Bu_4NI (2.5 mol%) and 0.7 g K_2CO_3 (5 mol%). After reaction at room temperature for 12 h, the pressure was released. 95% Yield of **2** was observed by GC and to the mixture another portion of **1** (8.5 g) was added followed by re-pressurizing air and CO for 12 h of reaction. Similarly, two more portions of **1** were added to the same reactor after a specific period of time. Altogether, the whole procedure was done corresponding to 0.033 mol% of catalyst loading. After crystallization, 30.9 g of **2** was obtained with 92% yield (See chart below).

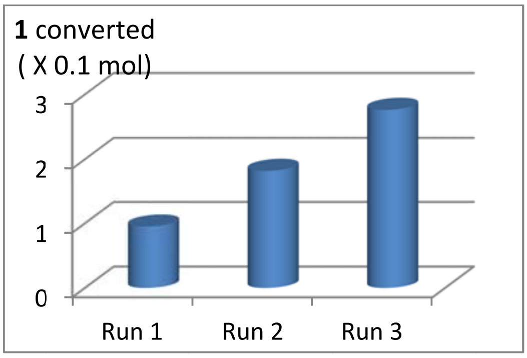

General procedure for Ru-catalyzed hydrogenation of oxamide to glycol

Under argon atmosphere, vial (4 mL) was charged with Ru catalyst, base, oxamide, and a stirring bar. Then, solvent was injected by the syringe. The vial was placed in an alloyed plate, which was then transferred into an autoclave (300 mL) under argon atmosphere. At room temperature the autoclave was flushed with H_2 three times, and then pressurized with H_2 to 60 bar. The reaction was performed at pre-set temperature for desired time. After the reaction finished, the autoclave was cooled to room temperature and the pressure was carefully released. The yield of ethyl glycol was measured by ^{1}H NMR analysis using tert-butanol as internal standard.

Synthesis and characterization of Fe-complexes

1) Synthesis of {$FeBr_2(CO)[HN((CH_2CH_2)P(CH_2CH_3))_2]$} **(c-8)**

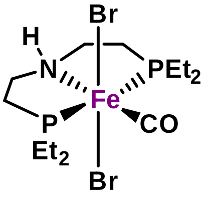

A solution of $HN(CH_2CH_2PEt_2)_2$ (250 mg, 1 mmol) in 2.5 mL of THF was added dropwise to a suspension of $FeBr_2{\cdot}2THF$ (0.375 g, 1.05 mmol) in 6 ml of EtOH. The resulting suspension was stirred at room temperature for overnight. Then the argon atmosphere replaced by carbon monoxide. A clear solution of blue color observed in 15 mins . Upon removal of the solvent, the residue was washed with EtOH (3 x 10 mL) to remove slight excess of unreacted $FeBr_2{\cdot}2THF$. Blue solid was obtained and dried under vaccum. **Yield: 330 mg** (68%).

Crystals were grown by the layer of THF and heptane.

^{1}H NMR (400 MHz, THF-d_8) 1.27-1.33 (m, 12H, P-(C$\underline{H}_3$)$_4$), 2.08-2.28 (overlapping m, 14H, P-(C$\underline{H}_2$)$_4$, P-C$\underline{H}_2$-), 3.15-3.24 (m, 2H, N-C$\underline{H}_2$), 3.43 – 3.50 (m, 2H, N-C$\underline{H}_2$), 4.98 (bt, 1H, NH); ^{13}C NMR (101 MHz, THF-d8, 297 k): δ 7.57 (s, P(CH_3)$_2$), 7.76 (s, P(CH_3)$_2$), 15.86-16.27 (m,

P*C*H_2), 27.81 (t, *J*C-P = 8.7 Hz, P-*C*H2), 49.51 (t, *J*C-P = 4.8 Hz, N*C*H_2), 225.38 (s, CO) ; ^{31}P NMR (162 MHz, THF-d8, 297 K) δ = 65.48 (s).
IR ATR: ῡ [cm-1] 1936 (s, ῡ CO)

2) Synthesis of {Fe(H)(HBH_3)(CO)[HN($CH_2CH_2P(CH_2CH_3)_2)_2$]}(**c-9**)

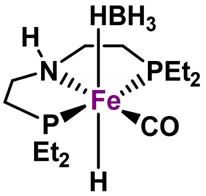

{{$FeBr_2$(CO)[HN((CH_2CH_2)P(CH_2CH_3))$_2$]} (330 mg, 0.67 mmol) was suspended in THF (10 mL) and $NaBH_4$ (256 mg, 6.7 mmol) in EtOH (5 mL) was added at room temperature. The reaction mixture was stirred for 2 h at room temperature to produce a bright yellow solution. The solvent was removed *in vacuo* and toluene (10 mL) was added. The resulting suspension was filtered and the liquid portion was concentrated *in vacuo*. The resulting solid was repeatedly washed with *n*-heptane to afford the title compound as a bright yellow solid. **Yield: 131 mg** (56%).
^{1}H{^{31}P} NMR (400 MHz, C_6D_6, 297 K) major isomer δ = -19.67 (s, 1H, Fe<u>H</u>), minor isomer δ = -20.30 (s, 1H, Fe<u>H</u>), -3.23 (br, FeB<u>H</u>$_4$, 4H), 0.98 (t, *J* = 7.6 Hz, 6H, P($CH_2C\underline{H}_3)_2$), 1.19 (t, *J* = 7.6 Hz, 6H, P($CH_2C\underline{H}_3)_2$), 1.34 – 1.59 (overlapping m, 8H, P($C\underline{H}_2CH_3)_2$, P$C\underline{H}_2$, N$C\underline{H}_2$), 1.67 (m, 2H, P$C\underline{H}_2$), 1.93 (m, 2H, P($C\underline{H}_2CH_3$), 2.20 (m, 2H, P($C\underline{H}_2CH_3$), 2.46 (m, 2H, N$C\underline{H}_2$), 3.78 (bt, 1H, N<u>H</u>); **^{13}C NMR** (101 MHz, C_6D_6, 297 K): δ 8.5 (s, P($CH_2\underline{C}H_3)_2$), 9.0 (s, P($CH_2\underline{C}H_3)_2$), 20.2 (t, $J_{C\text{-}P}$ = 11.2 Hz, P($\underline{C}H_2CH_3)_2$), 23.2 (t, $J_{C\text{-}P}$ = 13.7 Hz, P($\underline{C}H_2CH_3)_2$), 28.0 (t, $J_{C\text{-}P}$ = 8.3 Hz, P$\underline{C}H_2$), 53.3 (t, $J_{C\text{-}P}$ = 6.2 Hz, N$\underline{C}H_2$), 221.1 (<u>C</u>O); **^{31}P{^{1}H} NMR** (162 MHz, C_6D_6, 298 K) major isomer δ 81.8; minor isomer δ 78.8. **IR ATR** (solid): ῡ [cm^{-1}] 2358 (b, ῡ BH), 1898 (s, ῡ CO). **ESI-HRMS** (*m/z pos*): Calculated for [$C_{13}H_{33}BNOP_2Fe$]: 348.14771; found: 348.14804. **Elemental Analysis**: Anal. Calculated for [$C_{13}H_{34}BNOP_2Fe$]: C, 44.74; H, 9.82; N, 4.01. found: C, 44.61; H, 9.05; N, 3.75.

Oxamide-mediated production of EG from CO via two steps

Step 1: In a 300 mL autoclave, 6.1 mg Pd(acac)$_2$ (0.02 mmol) and 244 mg P(*o*-tol)$_3$ (0.8 mmol) were dissolved in 100 mL THF. After the solution was stirred at room temperature for 10 min, 1.48 g ^{n}Bu$_4$NI (4 mmol), 2.76 g K_2CO_3 (20 mmol), and 5.0 mL piperidine (50 mmol) were added. The autoclave was pressurized with air (25 bar) and CO (25 bar). After the reaction was stirred at room temperature for 16 h, the pressure was released. 99% yield of the corresponding oxamide **2** was observed by GC. Another portion of 5 mL piperidine **1** (50 mmol) was added into the mixture followed by re-pressurizing air (25 bar) and CO (25 bar). The reaction mixture was stirred at room temperature for 16 h. Similarly, two more portions of 5 mL piperidine **1** (50 mmol) were added to the same reactor after 16 h. Altogether, the whole procedure was done corresponding to 0.01 mol% of palladium catalyst loading. After all, 98% yield of the desired product was detected by GC analysis using isooctane as the internal standard.

Step 2: THF was removed from 60 mL reaction mixture of **step 1** followed by addition of 120 mL toluene. After stirring at room temperature for 20 min, the resulted mixture was filtrated through thin silica gel (2-3 cm). 5 mL toluene was used to wash the residue. The concentration of oxamide **2** in toluene was determined as follows: 1 mL filtrate was taken out followed by addition of 100 □L isooctane (internal standard), 94 mg oxamide **2** (0.42 mmol) in 1 mL toluene was observed by GC analysis. Then 12 mL toluene solution of **2** (5 mmol) was added into a 100 mL autoclave which charged with 29 mg Ru-MACHO-BH (0.05 mmol) and 11.2 mg KO^{t}Bu (0.1 mmol) under argon atmosphere. 8 mL toluene was injected by syringe to the autoclave. At room temperature the autoclave was flushed with H_2 three times, and then pressurized with H_2 to 60 bar. The reaction was performed at 160 ^oC for 12 h. Full conversion of **2** was detected by GC analysis. 96% yield of ethyl glycol was measured by ^1H NMR analysis using *tert*-butanol as internal standard.
